# Supplementary material for: Living restricted lives ‐ Understanding the impact of isolation, social distancing and other restriction measures on older care home residents and their relatives in England during the COVID-19 pandemic: A qualitative study
Source: PLoS One. 2024 Dec 4;19(12):e0312509. doi: 10.1371/journal.pone.0312509 (PMC11616855; doi:10.1371/journal.pone.0312509)
Supplement: S1 File — (DOCX) [file pone.0312509.s001.docx]

**S1 Interview guide for residents**

# **Study title: Protecting older people living in care homes from COVID-19: challenges and solutions to implementing social distancing and isolation**

## **Introduction**

Thank you again for helping us with our research. To remind you, our study is about social distancing and isolation in care homes during the coronavirus pandemic. When we are talking about social distancing and isolation, we mean staying a safe distance from other people to stop the virus from spreading. We want to find out how this has affected people who live and work in care homes.

I am going to ask you some questions about your experiences and views. You do not have to answer any questions that you do not want to. If you would like to stop at any time please let me know, you do not have to give a reason.

Do you have any questions before I begin?

If you are happy to start, then I will begin the tape and video [delete as appropriate] recording.

1. How were you told about the coronavirus?
   1. Do you feel that you know as much as you want to?
2. Have there been any changes in the care home since the coronavirus pandemic started?
   1. What sort of changes?
   2. How were you told about these changes?
   3. Do the changes make you feel safe?
3. Have there been any limits to where you are able to go in the care home?

[prompt if yes: where haven’t you been able to go? Have you been able to go into the garden? Explore if resident has been able to go to the communal lounge e.g. for activities? And to the communal dining room]

- 1. How have you felt about this?

1. Have there been any limits to whom you are allowed to see?

[prompt if yes: who have you been able to see or not see? Have you been able to see your friends who also live at the care home- how has this been managed? Have you been able to have family or friends visit- explore views and experiences of visiting arrangements during the pandemic e.g. new approaches such as window visiting, garden pods? Have you been able to see the doctor or other health professionals?]

- 1. How have you felt about this?

1. Have you spent more time on your own than you would normally?
   1. How do you feel about this? (Explore if confined to own room during the pandemic, what they were told about this. Explore how they passed their time when having to be in their own room)
2. Have any of the changes affected the care you receive?

[prompt if yes: in what way?]

1. Has your health changed since the coronavirus pandemic started?

[prompt if yes: in what way? explore physical and mental health]

1. What do you think the care home could do differently to help look after you during the coronavirus pandemic?

## Any other questions and thank you

Those are all my questions. Is there anything else you would like to say?

What will happen now is that we will send the tape recording to be transcribed, but we will not send any other information like your name or the name of the care home.

Once we have our final results, we will send you some information about what we found, if you gave us permission to do so.

Thank you very much for taking part.
