# Supplementary material for: Living restricted lives ‐ Understanding the impact of isolation, social distancing and other restriction measures on older care home residents and their relatives in England during the COVID-19 pandemic: A qualitative study
Source: PLoS One. 2024 Dec 4;19(12):e0312509. doi: 10.1371/journal.pone.0312509 (PMC11616855; doi:10.1371/journal.pone.0312509)
Supplement: S2 File — (DOCX) [file pone.0312509.s002.docx]

**S2 Interview guide for family members**

**Study title: Protecting older people living in care homes from COVID-19: challenges and solutions to implementing social distancing and isolation**

## **Introduction**

Thank you again for helping us with our research. To remind you, the aim of our study is to explore and understand the real-life experiences of social distancing and isolation of residents in care homes for older people, during the coronavirus pandemic. When we are talking about social distancing and isolation, we mean staying a safe distance from other people to stop the virus from spreading. We want to develop a variety of resources and guidance to support care homes throughout the coronavirus pandemic.

Sharing your views and experiences will help us to understand how the care home that your family member/friend [insert name of person if known] lives in has changed to meet social distancing and isolation regulations, and explore what has worked well and what hasn’t. This will be used to create the toolkit based on real-life examples.

I am going to ask you some questions about your experiences and views. You do not have to answer any questions that you do not want to. If you would like to stop at any time please let me know, you do not have to give a reason.

Do you have any questions before I begin?

If you are happy to start, then I will begin the recording.

1. What have been the restrictions to being able to visit your family member/friend [insert name of person if known] in the care home?
   1. How have you felt about that? (Explore views and experiences of new/different visiting arrangements during the pandemic)
   2. Has your wellbeing been affected? If so, in what way?
2. How is your family member/friend [insert name of person] adapting to the changes?
3. What has been the impact on your family member/friend [insert name of person]?
   1. Has their wellbeing been affected? If so, in what way?
   2. Has their health or physical wellbeing been affected? If so, in what way? (Explore views re impact on physical and mental well-being of resident)
4. How has the care home supported residents and their families/friends? (Explore views about what they think has/hasn’t worked well and why)
   1. Is there anything else you would have liked their support with?
   2. Is there any other support they could offer your family member/friend [insert name of person]
5. What could be done better/differently in the future?

## Any other questions and thank you

I have no more questions for you.

Is there any other information regarding your views on protecting older people in care homes from COVID-19 that you think would be useful for us to know?

What will happen now is that the recorded audio file will be sent to an external transcriber known to Kings College London, which complies with the Data Protection Act 2018 and GDPR. We will not send any identifying details with the audio file beyond what is said in the interview.

Once we have our final results, we will contact you to share them with you if you gave us permission to do so.

Thank you very much for taking part.
