# Supplementary material for: Living restricted lives ‐ Understanding the impact of isolation, social distancing and other restriction measures on older care home residents and their relatives in England during the COVID-19 pandemic: A qualitative study
Source: PLoS One. 2024 Dec 4;19(12):e0312509. doi: 10.1371/journal.pone.0312509 (PMC11616855; doi:10.1371/journal.pone.0312509)
Supplement: Attachment — Submitted filename: Reviewer PONE-D-23-08897.docx [file pone.0312509.s003.docx]

Paper Title: *Living restricted lives: understanding the impact of isolation and restriction on care home residents and their relatives in England during the COVID-19 pandemic*

Journal Manuscript ID: PONE-D-23-08897

**REVIEW**

This paper focuses upon the direct experiences of residents and their relatives of living restricted lives during the pandemic.

I think that the focus of this study could be interesting. Congratulations for your work and effort. However, I would like you to pay attention to the following indications for improvement its importance.

**Abstract:**

- Title: Indicate the study’s design with a commonly used term in the title
- Also, indicate the study design in the abstract
- To facilitate the reader's understanding, I recommend that typically includes background, purpose, methods, results, and conclusions.

**Methodology**

Information provided in the methods and procedures is not detailed or clear enough to replicate the study. I recommend the use of epigraphs for better understanding. See below for suggestions/ feedback.  Also, we strongly recommend you to use the *Consolidated Criteria for Reporting Qualitative Research guidelines* and

*The Working Group on Journal Article Reporting Standards for Qualitative Research*.

- Please, begin this section with Theoretical framework, Qualitative approach and Research paradigm: What methodological orientation was stated to underpin the study? e.g. grounded theory, discourse analysis, phenomenology, content analysis. Qualitative approach (e.g., ethnography, grounded theory, case study, phenomenology, narrative research) and guiding theory if appropriate; identifying the research paradigm (e.g., postpositivist, constructivist/ interpretivist) is also recommended; rationale.
- Sampling strategy - How and why research participants, documents, or events were selected; criteria for deciding when no further sampling was necessary (e.g., sampling saturation); rationale. In this section clarify the following issues and not in another section.
  - How were participants selected? e.g. purposive, convenience, consecutive, snowball.
  - How many people refused to participate or dropped out? Reasons?
- Data collection methods: Types of data collected; details of data collection procedures including (as appropriate) start and stop dates of data collection and analysis, iterative process, triangulation of sources/methods, and modification of procedures in response to evolving study findings.
- Add headings such as *Data analysis and rigor,* *Techniques to enhance trustworthiness.*

**Results**

- Make the topic heading smaller than the Results heading, so that you can see that it is in a lower category.

**Discusión**

- In my opinion, there should be more scientific discussion with more references to scientific articles.
