# Supplementary material for: Living restricted lives ‐ Understanding the impact of isolation, social distancing and other restriction measures on older care home residents and their relatives in England during the COVID-19 pandemic: A qualitative study
Source: PLoS One. 2024 Dec 4;19(12):e0312509. doi: 10.1371/journal.pone.0312509 (PMC11616855; doi:10.1371/journal.pone.0312509)
Supplement: Attachment — Submitted filename: Response to reviewers table submitted 080923.docx [file pone.0312509.s004.docx]

**PONE-D-23-08897	Response to reviewers’ feedback**

| **Recommendations from reviewer 1** | **Response** | **Page number** |
| --- | --- | --- |
|  |  |  |
| **Title:** Indicate the study’s design with a commonly used term in the title | Thank you, we have amended the study title to, ‘Living restricted lives - Understanding the impact of isolation, social distancing, and other restriction measures on older care home residents and their relatives in England during the COVID-19 pandemic: a qualitative study.’  We have revised the short title to: “The impact of restriction measures on older care home residents and their relatives.” | 1 |
| **Abstract:** To facilitate the reader's understanding, I recommend that typically includes background, purpose, methods, results, and conclusions. | Thank you, we followed the journal guidelines for the abstract, which did not suggest this format. However, we have now included these headings in the abstract, as requested. | 2 |
| **Methodology:** Information provided in the methods and procedures is not detailed or clear enough to replicate the study. I recommend the use of epigraphs for better understanding | Thank you, we have added sub-headers to organise the content in the 'Design and Methods' section and have expanded upon this. | 5 |
| **Methodology:** We strongly recommend you to use the *Consolidated Criteria for Reporting Qualitative Research guidelines* and *The Working Group on Journal Article Reporting Standards for Qualitative Research*. | Thank you, we had used the COREQ guidance for reporting qualitative research and have now made this clearer in the design section. We are not aware of *The Working Group on Journal Article Reporting Standards for Qualitative Research* but feel that using COREQ checklist is sufficient. | 5 |
| **Methodology:** please, begin this section with Theoretical framework, Qualitative approach and Research paradigm: What methodological orientation was stated to underpin the study? e.g. grounded theory, discourse analysis, phenomenology, content analysis. Qualitative approach (e.g., ethnography, grounded theory, case study, phenomenology, narrative research) and guiding theory if appropriate; identifying the research paradigm (e.g., postpositivist, constructivist/ interpretivist) is also recommended; rationale. | Thank you, we have added a ‘Design’ section to ‘Materials and Methods’ to make clear that the qualitative study reported in this paper was conducted as part of a mixed -methods, phased study. | 5 |
| **Methodology:** Sampling strategy - How and why research participants, documents, or events were selected; criteria for deciding when no further sampling was necessary (e.g., sampling saturation); rationale. In this section clarify the following issues and not in another section. How were participants selected? e.g. purposive, convenience, consecutive, snowball. How many people refused to participate or dropped out? Reasons? | Thank you, further detail about how care homes, residents and relatives were sampled and recruited has been presented in the respective sections ‘Setting’ and ‘Participants’. We have reported that we were only able to recruit two family members at care home 5, and at care home 6 we were only able to recruit two residents who had capacity to consent.  Characteristics of participating residents and relatives are presented in Table 1. | 5-6 |
| **Methodology:** Data collection methods: Types of data collected; details of data collection procedures including (as appropriate) start and stop dates of data collection and analysis, iterative process, triangulation of sources/methods, and modification of procedures in response to evolving study findings. | Thank you, we have reported further detail about our data collection methods i.e., individual interviews with residents and family members. We have reported that interviews at care homes were conducted non-simultaneously, and interviews at one care home were generally completed before interviews at another care home began. This sequential approach to data collection meant that data were collected at different times for different care homes. We have reported that additional prompts were added to the schedules following the initial interviews in the first care home and were agreed upon by the research team. We have reported on the demographic data that were collected for participating residents and relatives. | 6-9 |
| **Methodology:** Add headings such as *Data analysis and rigor,* *Techniques to enhance trustworthiness.* | Thank you, to make transparent our approach to enhancing rigour and trustworthiness, we have added further detail in the ‘Data analysis’ section, e.g., reporting that all interviewers are established academic researchers with experience in qualitative interviewing and a background in health and/or social care research. We have reported that audio recordings were transcribed verbatim by an approved transcribing company and were quality assured by a researcher.  In addition, we reported that analysis was led by two researchers (blind for review), though a sub-section of the transcripts were also read and analysed by other members of the research team (blind for review) to ensure consistency of approach and provide quality assurance. | 9 |
| **Results:** Make the topic heading smaller than the Results heading, so that you can see that it is in a lower category. | Thank you, we have ensured throughout that topic headings are now smaller than section headings. | Throughout |
| **Discussion:** In my opinion, there should be more scientific discussion with more references to scientific articles. | Thank you, we have developed further our Discussion and have incorporated further supporting literature. | 19 |
|  |  |  |
| **Recommendations from reviewer 2** | **Response** |  |
| **Methodology:** How were care home contacted. You say they were nominated by a provider so I wonder did you have variety of provider? | Thank you, we have added additional information to the ‘Setting’ section to make this clearer. | 5 |
| **Methodology:** Recruitment - I like the idea of a project champion especially in care home research where recruitment can be very challenging. I wonder how capacity to give consent was assessed and were their residents who expressed an interest but were found not to have capacity.  There should be mention of limitations of sampling which was through care home gatekeepers, Even though I fully accept this was the most practical way during the pandemic. | Thank you, as stated in the ‘Participants’ section, inclusion criteria were that residents must be over 65 years old and have the capacity to consent. Residents who met the criteria were identified and nominated by the care home manager and were introduced to the study by the project champion.  Thank you, this limitation has now been added to the paper, along with the limitation that all care homes were rated by the CQC as either ‘outstanding’ or ‘good’. | 6  25 |
| **Discussion:** A little more nuanced consideration of the results would improve the discussion. For example page 15 line 320 states resident and relatives appreciates staff efforts for purposeful entertainment yet the results identifies that poor mobility could hinder residents involvement. I wonder were there any themes of inequity coming through the data. I was also struck that some residents could independently use video or phone technology but others relied on staff. The published protocol paper for this study mentioned the toolkit it might be helpful if the final part of the discussion makes readers of this paper aware of next steps. | Thank you, we have developed further our Discussion and have incorporated further supporting literature. We have included in our implications of the study findings, that a toolkit for care homes has been developed. | 19 |
| **Conclusion:** Please review the conclusion the first sentence is not clear ‘the loss of older people’ is this we ‘lost older people’ relating to statistics on deaths, or the older people experienced this type of loss due to ..’ | Thank you, this sentence has been amended to make clear that we are referring to statistics on deaths of older people living in care homes. | 26 |
