# Supplementary material for: Living restricted lives ‐ Understanding the impact of isolation, social distancing and other restriction measures on older care home residents and their relatives in England during the COVID-19 pandemic: A qualitative study
Source: PLoS One. 2024 Dec 4;19(12):e0312509. doi: 10.1371/journal.pone.0312509 (PMC11616855; doi:10.1371/journal.pone.0312509)
Supplement: Attachment — Submitted filename: PONE-D-23-08897R2-Response to reviewers.docx [file pone.0312509.s005.docx]

**PONE-D-23-08897R2**

**Living restricted lives - Understanding the impact of isolation, social distancing and other restriction measures on older care home residents and their relatives in England during the COVID-19 pandemic: a qualitative study.**

**Response to reviewers’ comments received 31 May 2024**

| **Reviewer comment** | **Response** | **Page number** |
| --- | --- | --- |
| The [PLOS Data policy](https://eur03.safelinks.protection.outlook.com/?url=http%3A%2F%2Fwww.plosone.org%2Fstatic%2Fpolicies.action%23sharing&data=05%7C02%7Csarah.sims%40kcl.ac.uk%7C3f201b913fcc4991faf008dc8415b0dd%7C8370cf1416f34c16b83c724071654356%7C0%7C0%7C638530475908004367%7CUnknown%7CTWFpbGZsb3d8eyJWIjoiMC4wLjAwMDAiLCJQIjoiV2luMzIiLCJBTiI6Ik1haWwiLCJXVCI6Mn0%3D%7C0%7C%7C%7C&sdata=GV37iRExB1rqMoFr075oM8MdnXYg8gIIQgLkyJ5yj6A%3D&reserved=0) requires authors to make all data underlying the findings described in their manuscript fully available without restriction, with rare exception (please refer to the Data Availability Statement in the manuscript PDF file). The data should be provided as part of the manuscript or its supporting information, or deposited to a public repository. For example, in addition to summary statistics, the data points behind means, medians and variance measures should be available. If there are restrictions on publicly sharing data—e.g. participant privacy or use of data from a third party—those must be specified. | Thank you for your comment. We have expanded on this statement to reflect the PLOS Data Policy. | 28 |
| Reviewer #3 - The study is motivated by "an understanding of the physical and psychological impacts of the restrictions imposed upon care home residents" being limited. Many studies have explored the physical and psychological impacts of pandemic restrictions. A more detailed discussion of how your study builds upon, diverges from, or fills gaps in this literature would highlight its scientific value and theoretical implications more clearly. Here, the cited literature seems to be a bit thin, as there are several studies in the record now that have explored this and related issues (such as how technology for social interaction was put to use during the pandemic). I would like to see your stated motivation for the paper more clearly engaging with this research record. | There have been some additional studies published since we submitted our paper to PLOS-ONE over 15 months ago. We have incorporated additional studies into the revised manuscript at various points.  There have been many studies that have explored the physical and psychological impacts of the pandemic restrictions. However, very few have explored this with residents of care homes and their families and the literature remains thin.  Even fewer studies were undertaken at the time the restrictions were in force because of the challenges in undertaking research at that time. We believe our study provides a rare insight into the experiences and perspectives of one of the most vulnerable groups as they were living with the restrictions. These restrictions have particular significance given the life expectancy of care home residents. | 4  Several pages in the Discussion section starting on page 20. |
| Reviewer #3 - I agree that such experiences are worthwhile documenting, but what is the scientific value of describing them (besides bringing them into the light)? Do the authors see any particular implications from this knowledge? Are there conceptual or theoretically interesting consequences from this knowledge? In the revised version, the authors give a high level description of how the findings can inform discussions, guideline development etc. at the end. This strikes me as a missed opportunity, and can be used to better motivate the article (and the problem space it is addressing) up front. In other words, the article needs to better answer the "so what" question. | Thank you for your support of documenting the experiences of residents and their families during the COVID-19 pandemic.  The implication of this knowledge is that it is rare.  Very few studies have captured the experience of residents, who were a hard-to-reach group. Understanding their perspectives is very important as their views are not what you might expect and can inform any future pandemics. Our aim in this paper was to report their experiences in detail. We have added some text to clarify this. | 4,  Discussion from page 20 to 27.  Conclusions from page 27 to 28. |
| Reviewer #3 - One of the former reviewers asked for a better grounding in terms of research design, which I feel is not fully addressed yet. The revised version contains a section on design, where it is clear that this study emerges from Phase 2. However, that phase also mentioned a lot of other materials, in addition to the interviews here described. What is the relationship between this material, and the other aspects of the study (cursory mention is made of the "toolkit", but the reader does not really get a good grasp of what this is. There is additional qualitative materials that seems relevant from Phase 2, which is not engaged with here. Further clarification on the research design, particularly the relationship between the interviews conducted and other materials from Phase 2 of your study, would enrich the reader's understanding. Describing the toolkit and additional qualitative materials mentioned would offer a more comprehensive view of your research approach | Thank you for your insightful comment.  The detailed research proposal and design has been published and we have clarified this in the paper. | 5 |
| Reviewer #3 - Moreover, what is meant by "direct experiences" by the authors? This appears to be a potentially theoretically loaded concept, but this use is not situated within a broader stream of literature. I assume the authors aim to describe some sort of first-person experiences about the pandemic restrictions - but they do not provide any kind of theoretical or conceptual grounding of these experiences (e.g., narrative research, phenomenology, etc.) - I don't have a particular conceptual axe to grind here, but there is definitely literature that could be engaged with here to make the paper more interesting. Without a grounding of this kind, it is a bit difficult to assess the kind of experiential claims being reported, as the qualitative material - e.g. use of quotations- is are fairly modest (probably due to restrictions on space). | Residents experiences of living in a care home during the COVID-19 pandemic are often described by others e.g. care home staff or their relatives. When we said ‘direct experiences’ we meant that we interviewed residents themselves. It wasn’t a theoretically loaded concept therefore we have deleted ‘direct’ to avoid confusion.  We have added further quotations from residents. | 5,  Results section from page 11 to page 20. |
| Reviewer #3 - I am wondering to what extent the themes identified by the authors reflect the interview guide, which is not uncommon in interview-based studies of this kind. How did the interview guide look? What is the relationship between the themes, and the structure of the interview guide? How many codes did the authors encounter, what where the coding groups you ended up with (are the ones presented here exhaustive, etc.)? A table would be helpful, alongside more information about what the content of the interviews revolved around. The best qualitative research - based on thick descriptions - gets us beyond the themes that can be predicted in advance - based on the interview guide for instance. More clearly showing the relationship between these themes and your codes might add further depth. | We have included the interview guides for residents and relatives as supplementary material.  Analysis was conducted by a team of experienced healthcare researchers- we have expanded on this in the paper. | The two interview schedules are presented as Supporting information 1.  Further detail on data analysis is presented on page 10. |
| Reviewer #3 - The conclusion could be stronger - 'these findings need to be considered to fully understand the experience of care home residents and their relatives of living restricted lives during the COVID-19 pandemic'. Again, so what? What are the general insights of interest here, for caring sciences or health services research? The revised version is more explicit about this issue, but there is still potential to draw this out more clearly. The toolkit that was developed as part of the project, for instance, might hold some relevance here. The conclusion could be strengthened by articulating the general insights of your findings more explicitly and discussing their relevance to caring sciences or health services research. Mentioning the toolkit developed as part of the project and its potential applications could provide a compelling case. | Thank you for your helpful comments. We have taken these on board to develop further the conclusions. | 27-28 |
| Reviewer #3 - Finally, considering the specific insights and implications of your study, help clarify the intended audience for this contribution. | Thank you. We agree that a fuller consideration of the rich insights from residents and relatives and the implications of the study have helped to clarify the intended audience. | Discussion from pages 20 to 27.  Conclusions on pages 27 & 28. |
| Reviewer #4 - Abstract Please indicate in methods if all resident participants were older adults. | All resident participants were older adults and this has now been indicated in the Abstract. | 2 |
| Reviewer #4 - Introduction Pg. 3, line 48: Please briefly define what is meant by “social care”, this is not a universally understood model of care. There also needs to be a description of how this care model is funded.  It would also be helpful for the authors to clarify who the residents living in social care are. For example, do the majority have dementia? How extensive are their care needs? | A description of social care in the United Kingdom including care homes, has been included. We have also made more explicit the needs of older adults living in UK care homes, and how living in care homes is usually funded. | 3-5 |
| Reviewer #4 - Pg. 3, line 55: Please clarify context. Are older adults at risk of poor outcomes if they are vaccinated against COVID-19. If authors are referring to pre-vaccine times, they need to clarify. | We have added text surrounding the context of the COVID-19 vaccination programme. | 3-4 |
| Reviewer #4 - Pg. 3, line 56: Please provide an exact date for the statistic of 16.7%. Suggest authors briefly discuss the fact that the swift and high mortality rates associated with COVID-19 in the early waves necessitated drastic measures (e.g., isolation) despite little research existing for their potential collateral impacts on social/mental well-being. Context is very important for understanding this research study. | Specific dates for the 16.7% statistic are presented in the manuscript.  More information about the context which necessitated the implementation of the restrictive measures has been provided. | 4,5 |
| Reviewer #4 - Pg. 5, line 89: Please clarify what the film was for. | Thank you, details of the purpose of the film have been added. | 6 |
| Reviewer #4 - Materials and Methods Please provide exact dates when this study was executed at the beginning of the methods section. Who determined whether residents had the capacity to consent? Please clarify. Did residents require support to use the iPad or was this part of the inclusion criteria that they be able to manage the device alone. How were questions for qualitative interviews developed? Were they informed by a theoretical framework or model? Please include full interview guide as an appendix. More information is required regarding the case study approach utilized by the authors. What are the methodological underpinnings of this approach? Please provide more information including appropriate references.  Methods section would be strengthened by discussing the authors positionality with regards to the research and discussing how they maintained reflexivity throughout the study processes.  More information required about how study rigor was ensured required. Using quotes from different participants is standard within qualitative research, this in and of itself doesn’t necessarily enhance the trustworthiness of the data. | -Dates for data collection have been included at the start of the ‘setting’ section.  -We have clarified that it was the care home manager who advised us regarding residents’ capacity to consent. We have also included further information about the process consent framework.  -Information on support made available to residents to use the iPad is presented, we have expanded on this.  -More information on the interview schedule has been added and we have included as supplementary material the interview schedules for residents and relatives.  -More information about the case study approach has been provided.  - Strategies to maintain reflexivity and trustworthiness have been presented more fully. | Materials and Methods section, pages 5 to 11. |
| Reviewer #4 - Results Did the authors collect information on other genders or only male/female as options? Please provide a brief statement at the beginning of the results section stating what the main themes are prior to discussing them. Quotes should be separated by text not piggy backed onto one another within the results. | -Gender identity was stated by the participant and this has now been included in the paper.  -A short statement at the beginning of the results section highlighting the main themes has been added.  -All quotes have now been separated by text. | 12,  Results section from page 11 to page 20. |
| Reviewer #4 - Pg. 12 & 13: large section of description without quotes to support these findings. More exemplars from interviews required to make these claims. Pg. 16: more exemplar quotes require to support perspectives made by authors. | -New quotes have been included. | Results section from pages 11 to page 20. |
| Reviewer #4 - Discussion This section could be strengthened with inclusion of more literature particularly in section entitled isolation measures. There is a growing body of literature that speaks to the impacts of social isolation but also resilience among this population. | Thank you for your feedback. We have developed further the Discussion to encompass the literature referred to by reviewer 3, point 2. | Discussion from pages 20 to 27. |
| Reviewer #4 - Strength and Limitations Pg. 24, line 515: This is not a strength, since interviews were cross-sectional and member checking was not done. Therefore the participants were not retained over time. | This has been changed to ‘recruited and interviewed’ rather than ‘recruited and retained’. | 26 |
| Reviewer #4 - The authors should not discuss the toolkit in the conclusion as it is not discussed anywhere else in the manuscript. | References to the toolkit have been removed. |  |
| Reviewer #4 - Please review manuscript for grammatical areas (multiple periods) and repetitive language e.g., last paragraph use “our findings” three times in close succession. | This has been completed and changes to the text have been made in the last paragraph. | The whole manuscript has been reviewed and revisions have been made to the Conclusions on pages 27 & 28. |
